# Handling of dietary protein in critically ill patients

**DOI:** 10.1186/cc12181

**Published:** 2013-03-19

**Authors:** F Liebau, O Rooyackers, L Van Loon, J Wernerman

**Affiliations:** 1 Karolinska University Hospital Huddinge, Stockholm, Sweden; 2 NUtrIm, Maastricht University, Maastricht, the Netherlands

## Introduction

Protein turnover measurements by stable isotope techniques can be applied to assess the nutritional/metabolic status in critically ill patients and their response to feeding. Because of uncertain gastrointestinal transport and uptake of nutrients, their contribution needs to be measured separately. We studied whole-body protein kinetics, with special emphasis on the contribution of dietary protein, in ICU patients and healthy controls.

## Methods

Mechanically ventilated, not enterally fed ICU patients (*n = *9) were recruited from an interdisciplinary ICU. Healthy, overnight-fasted volunteers (*n = *6) served as reference. A primed constant i.v. infusion of ^2^H-labeled phenylalanine (Phe) and tyrosine was used to quantify whole-body protein metabolism. Patients remained on parenteral nutrition (PN) as clinically indicated; controls received PN starting 2.5 hours before starting enteral feeding. Intrinsically ^13^C-Phe-labeled casein was infused for 6 hours by nasogastric tube at 0.75 g protein/ hour, together with maltodextrin at 2.73 g/hour. Protein breakdown, synthesis, net balance, and Phe splanchnic extraction were calculated before and at the end of the enteral feeding period, using equations for steady-state whole-body protein kinetics. Comparisons were made by Wilcoxon matched pairs and Mann-Whitney U tests; values are reported as mean ± SD.

## Results

Protein net balance was lower in patients than in the reference group at baseline (-1.8 ± 1.7 vs. 0.6 ± 0.6 mg/kg BW/hour, *P *= 0.003), and after enteral feeding (-1.1 ± 1.5 vs. 0.6 ± 0.6 mg/kg BW/hour, *P *= 0.049). Recovery of labelled Phe from enteral feeding into the systemic circulation was higher in the reference group as compared with patients (20.3 + 11.2% vs. 7.0 + 4.8%, *P *= 0.005). Enteral feeding did not affect protein metabolism in the reference group. In patients, protein breakdown became slightly lower during enteral feeding (10.6 ± 3.3 vs. 11.2 ± 3.3 mg/kg BW/hour, *P *= 0.086) and protein net balance became slightly higher (-1.1 ± 1.5 vs. -1.8 ± 1.7 mg/kg BW/ hour, *P *= 0.086).

## Conclusion

Intrinsically isotope-labelled casein can be used to quantify dietary contribution to protein metabolism in critically ill patients. Hypocaloric enteral feeding marginally improved protein balance in these patients. The low recovery of enterally administered labelled amino acid underlines the need to quantify uptake from the gastrointestinal tract when protein turnover measurements are performed in critically ill patients on enteral nutrition.

